# Some like it, some do not: behavioral responses and central processing of olfactory–trigeminal mixture perception

**DOI:** 10.1007/s00429-020-02178-4

**Published:** 2020-12-23

**Authors:** Franziska S. Müschenich, Thorsten Sichtermann, Maria Elisa Di Francesco, Rea Rodriguez-Raecke, Lennart Heim, Marco Singer, Martin Wiesmann, Jessica Freiherr

**Affiliations:** 1 Diagnostic and Interventional Neuroradiology, University Hospital, RWTH Aachen University, Pauwelsstraße 30, 52074 Aachen, Germany; 2 Scent and Care, Symrise AG, Holzminden, Germany; 3 Department of Psychiatry and Psychotherapy, Friedrich-Alexander-University Erlangen-Nürnberg, Erlangen, Germany; 4 Sensory Analytics, Fraunhofer Institute for Process Engineering and Packaging IVV, Freising, Germany

**Keywords:** fMRI, Insula, Pain matrix, Somatosensory, Trigeminal enhancement

## Abstract

Exploring the potential of eucalyptol as a masking agent for aversive odors, we found that eucalyptol masks the olfactory but not the trigeminal sensation of ammonia in a previous study. Here, we further investigate the processing of a mixture consisting of eucalyptol and ammonia, two olfactory–trigeminal stimuli. We presented the two pure odors and a mixture thereof to 33 healthy participants. The nostrils were stimulated alternately (monorhinal application). We analyzed the behavioral ratings (intensity and pleasantness) and functional brain images. First, we replicated our previous finding that, within the mixture, the eucalyptol component suppressed the olfactory intensity of the ammonia component. Second, mixture pleasantness was rated differently by participants depending on which component dominated their mixture perception. Approximately half of the volunteers rated the eucalyptol component as more intense and evaluated the mixture as pleasant (pleasant group). The other half rated the ammonia component as more intense and evaluated the mixture as unpleasant (unpleasant group). Third, these individual differences were also found in functional imaging data. Contrasting the mixture either to eucalyptol or to both single odors, neural activation was found in the unpleasant group only. Activation in the anterior insula and SII was interpreted as evidence for an attentional shift towards the potentially threatening mixture component ammonia and for trigeminal enhancement. In addition to insula and SII, further regions of the pain matrix were involved when assessing all participant responses to the mixture. Both a painful sensation and an attentional shift towards the unpleasant mixture component complicates the development of an efficient mask because a pleasant perception is an important requirement for malodor coverage.

**Electronic supplementary material:**

The online version of this article (10.1007/s00429-020-02178-4) contains supplementary material, which is available to authorized users.

## Introduction

We are rarely surrounded by pure olfactory or trigeminal stimuli but rather by bimodal olfactory–trigeminal mixtures. In our investigation of an efficient tool for malodor coverage, we, therefore, focus on masking behavior within a mixture consisting of two bimodal components. In a recent study, we proved that eucalyptol is an effective mask to cover an aversive odor: the fresh smell of eucalyptol masked the urine-like smell of ammonia, indicating potential industry applications such as improving the smell of hair dye or used animal litter. However, the trigeminal sensation of ammonia could not be reduced by eucalyptol, instead, the trigeminal sensation of the mixture was enhanced (Müschenich et al. 2019). In the present study, we repeated the experiment and focused on the underlying neural processing using fMRI. The aim was to investigate neural responses to the single bimodal stimuli and a mixture thereof in which the negative odor is supposed to be masked by the positive odor.

It is known that the two chemical systems interact closely and are based on overlapping processing circuits [for a review, see Albrecht et al. (2010), Boyle et al. (2007b), Han et al. (2018), Hummel et al. (2005) and Lundström et al. (2011)]. Previous studies reported mixture-related activations in the rostral insula, the orbitofrontal cortex (OFC), the superior frontal gyrus, the angular gyrus, and the middle cingulate gyrus (Boyle et al. 2007a, 2009; Hummel et al. 2013). However, there has been neither literature about mixing two bimodal stimuli nor about masking behavior.

Researcher found enhanced neural activation in response to a mixture (Boyle et al. 2007a, b, 2009) which is in line with our previous study reporting enhanced perceived mixture intensity on a behavioral level (Müschenich et al. 2019). Activation in cortical regions such as the cerebellum, the medial frontal gyrus, the piriform cortex (pirC), the entorhinal cortex, the anterior cingulate cortex (ACC), and the amygdala were associated with the processing of stimulus intensity (Anderson et al. 2003; Bensafi et al. 2008; Grabenhorst et al. 2007; Iannilli et al. 2011; Oertel et al. 2012; Rolls et al. 2003; Royet et al. 2000; Sobel et al. 1998).

Since higher stimulus intensity is usually correlated with less pleasantness, it must be precisely determined which neural activation represents which attribute. However, interactions can still remain undetected (Bensafi et al. 2008; Cometto-muñiz and Cain 1984; Distel et al. 1999). According to our study objective of malodor coverage, a pleasant mixture perception is an important condition. It was reported that perceived pleasantness did not differ between a mixture and its pleasant component despite the unpleasant component (Grabenhorst et al. 2007). Activations in the medial and rostral OFC, the anterior portion of the ACC, and the pirC were positively correlated with pleasantness and activations in the lateral and posterior OFC, the dorsal ACC, the anterior insula/frontal operculum, and the pirC were negatively correlated with pleasantness (Anderson et al. 2003; Grabenhorst et al. 2007; Rolls et al. 2003; Zelano et al. 2007). If pleasant and unpleasant stimuli were mixed, the mixture could cause cerebral activation in regions coding for pleasantness and unpleasantness while on a purely behavioral level only the pleasant percept was reported (Grabenhorst et al. 2007). We observed the latter in our previous study as well (Müschenich et al. 2019).

In the present study, we aim to (1) confirm our previous results on a behavioral level, (2) evaluate differences in the perceived pleasantness between single and mixed stimuli, and (3) explore cortical activation in response to a binary mixture compared to its single components. We hypothesize that the mixture will be rated as pleasant as eucalyptol and that neural activation patterns of these two stimuli will be very similar. Thereby, we could strengthen the role of eucalyptol as a masking tool.

## Methods

### Participants

Prior to participation, all subjects signed informed written consent. In total, 39 healthy volunteers took part. Six subjects had to be excluded from analysis due to various reasons (anatomical brain anomaly, inability to differentiate between the smell of eucalyptol and ammonia, and technical problems with image co-registration). The remaining group consisted of 33 right-handed non-smokers (20 females) who met the inclusion criteria: Ages ranged from 20 to 36 years [mean (*M*) = 25.7 years; standard deviation (SD) = 3.8 years] and the average body mass index (BMI) was 22.2 kg/m^2^ (SD = 2.1 kg/m^2^, range 18.8–25.7 kg/m^2^). Normosmia was assessed performing the MONEX-40 identification task [score: *M* = 33.5, SD = 2.4, range 28–40; cutoff: ≤ 27 (Freiherr et al. 2011)]. Participants were excluded if they took medication or suffered from diseases concerning the nose or lung. Beck’s Depression Inventory [BDI; score: *M* = 1.1, SD = 2.5, range 0–12; cutoff: ≤ 13 (Beck et al. 1961)], Brief Symptom Inventory [BSI; score: *M* = 40.1, SD = 6.2, range 27–54; cutoff: based on the age and gender-based SCL-90-R norm values with GSI ≤ 60 (Derogatis and Melisaratos 1983)], and Montreal Cognitive Assessment [MOCA (Paez-Venegas et al. 2018) score: *M* = 29.1, SD = 1.0, range 26–30; cutoff: ≥ 26] were performed to exclude psychiatric, psychological, and cognitive impairments. All participants were carefully controlled for MRI-specific exclusion criteria such as metal implants, retainers, tattoos, and piercings. This study is in accordance with the ethical standards of the institutional research committee of the medical faculty of RWTH Aachen University and with the 1964 Helsinki Declaration and its later amendments or comparable ethical standards.

### Stimuli

We used the following stimuli: 100% eucalyptol (Eucalyptol natural, Product No.: 130301, Symrise AG, Holzminden, Germany), 0.006% (60 ppm, parts per million) ammonia, a mixture thereof, and odorless propylene glycol (PG; 1,2-Propanediol, CAS: 57-55-6, Sigma-Aldrich, Steinheim, Germany) serving as a baseline. We prepared two stock solutions of 1.6 mol/L by dissolving 8.56 g ammonium chloride (NH_4_Cl, 99.5%; CAS: 12125-02-9, Sigma-Aldrich, Steinheim, Germany), respectively, 6.40 g sodium hydroxide (NaOH, 98%; CAS: 1310-73-2, Sigma-Aldrich, Steinheim, Germany) in 100-mL distilled water. The final ammonia solution consisted of 480-μL ammonium chloride, 960-μL sodium hydroxide, and 8.56-mL water. The mixture stimulus was generated within the olfactometer by combining the airflows of eucalyptol and ammonia in equal parts. Further details on stimulus preparation and the stimulus-dependent airflows within the olfactometer can be found in our previous work (Müschenich et al. 2019). We further reported that the mentioned stimulus concentrations were sufficient for our final study objective, namely to mask the smell of ammonia by eucalyptol. We showed that both pure odors affect the olfactory as well as the trigeminal system. By means of a photoionization detector (PID; 200B miniPID. Aurora Scientific Inc., Aurora, Ontario, Canada), we confirmed that the applied odor delivery system worked reliably. Results confirmed for each odorant that maximum and shape of the amplitude remain constant across time. For more details, see Müschenich et al. (2019).

### Stimulus presentation

A computer-controlled olfactometer which was calibrated to an air flow of 2 L/min (liter per minute) was used for odorant delivery (Lundström et al. 2010). The experimental setup was designed to maintain equal airflow in both pure and mixed conditions and is described in detail in Müschenich et al. (2019). The olfactometer was placed and operated in the control room. The odorants were delivered via Teflon tubes into the scanning room and into the participant’s nose by nose pieces fixed on the tubes. Stimuli were presented to one nostril (monorhinal) while PG was presented to the contralateral nostril. Every stimulus was prepared for the right and the left nostril resulting in eight experimental conditions (eucalyptol, ammonia, mixture, and PG, each for left and right nostril). The application duration was 4 s. PG presentation during the interstimulus-interval (ISI) provided a continuous airflow during the whole experiment and avoided neural activation changes due to airflow changes within the nose. The ISI was at least 30 s to prevent trigeminal and olfactory desensitization (Hummel and Kobal 1999). It was increased for each nostril since odorant delivery was alternated between nostrils and because of an implemented respiration-triggered olfactory stimulation method (RETROS) which is described elsewhere (Hoffmann-Hensel and Freiherr 2016). By means of RETROS, we avoided sniffing-related brain activation because the application of the next stimulus was continuously adjusted to the participant’s individual respiratory cycle. There was no cue before odor delivery which could have biased the subject’s odor perception.

### Experimental procedure

We divided the experiment into two identical functional imaging sessions lasting approximately 30 min each with a break of 45 min. Each session comprised ten trials per stimulus. This resulted in 80 trials in total including both sessions. The order of conditions was pseudorandomized which means that no odor was presented twice in a row and the odor stimulus was always delivered to alternate nostrils in successive trials. The experiment was programmed in Psychopy2 (Psychophysics software in Python; Peirce 2008) using an event-related design. Before starting the experiment, we presented eucalyptol and ammonia to the participants using the olfactometer for familiarization of the subjects with the stimuli.

The participants’ task was to rate intensity and pleasantness after each trial. More precisely, they were asked to rate eucalyptol component and ammonia component intensity, total intensity, as well as pleasantness on two visual analogue scales (VAS). The scale values ranged from 0 to 100 but the increments were not visible. Instead, participants read “not perceived” or “very unpleasant” at the beginning of the scale and “very intense” or “very pleasant” at the end of the scale. The marker was moved along the scale by the participants. To keep the number of questions low, participants rated neither trigeminal intensity nor trigeminal pleasantness. In addition, a differentiation of the two concepts would have forced us to train participants on the different percepts and how to rate one without considering the other quality. Since this was the first experiment employing those stimuli and we did not want to overwhelm and exhaust the subjects, we decided to only include the three questions per stimulus. Responses were submitted using an MRI-compatible keyboard placed under the participants right hand. Instructions and VAS were presented on a screen (NordicNeuroLab 40″ 4 K UHD InroomViewingDevice, NordicNeuroLab AS, Norway) positioned behind the scanner which was visible to the participants via a mirror integrated into the head coil.

### Data acquisition

All measurements were performed on a 3-T MRI scanner (Siemens Magnetom Prisma, Erlangen, Germany) with a 20-channel head-coil (Siemens, Erlangen, Germany). During functional imaging, whole-brain echoplanar images (EPI) were acquired with 36 slices (192 × 192 mm^2^ field of view (FOV), 80 × 80 matrix, 2.5 × 2.5 × 2.5 mm^3^ voxel size, 20% distance factor, 2-s repetition time (TR), 30-ms echo time (TE) and a 77° flip angle). The slice package was positioned on a sagittal localizer scan parallel to the AC–PC line (anterior commissure/posterior commissure). Further, a T1-weighted high-resolution structural image was acquired for each participant (MPRAGE: magnetization prepared rapid gradient-echo sequence with 1 × 1 × 1 mm^3^ voxel size, 256 × 256 mm^2^ FOV, 2-s TR, 2.28-ms TE and 8° flip angle, 4.40-min acquisition time). We recorded the respiration during the entire experiment using a PowerLab 8/35 device (ADInstruments Ltd., Dunedin, New Zealand) and LabChart 8 (ADInstruments Ltd., Dunedin, New Zealand) for data visualization. Due to technical problems during recording, we excluded the respiratory data of one participant (*n* = 32).

### Behavioral data analysis

All statistical calculations were performed using IBM SPSS Statistics software version 25 (Armonk, New York, United States). We analyzed the behavioral data running three univariate ANOVA with the dependent variables “intensity” (model I and model II) and “pleasantness” (model III). In each model, we estimated effect sizes by calculating partial eta-squares (*η*^2^). Subsequent post hoc tests were Bonferroni corrected and *p* < 0.05 was considered significant.

*Intensity ratings* In model I, “stimulus” (what was presented: eucalyptol, ammonia, or mixture), “target” (what was rated: eucalyptol component, ammonia component, or total intensity), and “session” (session 1 or session 2) were included as fixed factors. We focused on the interaction effect “stimulus” × “target” to assess whether intensity differences in the eucalyptol component between pure and mixed conditions reached significance compared to differences in the ammonia component between pure and mixed conditions.

To compare the intensity of the eucalyptol component in pure eucalyptol and the ammonia component in pure ammonia, we used model II. The fixed factors “stimulus type” (pure or mixed), “component” (rated component intensity: eucalyptol component or ammonia component), and “session” (session 1 or session 2) were included. Further, the interaction “stimulus type” × “component” was examined to compare the differences between the pure and mixed condition for the eucalyptol and ammonia component.

*Pleasantness ratings* Model III was run with the fixed factors “stimulus” (eucalyptol, ammonia, mixture, or PG) and “session” (session 1 or session 2).

To further explore the perceived mixture pleasantness, we grouped the participants into pleasant group, those who rated the mixture as more pleasant than PG, and unpleasant group, those who rated the mixture as less pleasant than PG. A paired sample *t* test was used to compare the groups in terms of their intensity ratings in the mixture condition (eucalyptol component intensity, ammonia component intensity, and total intensity). These intensities were then correlated with the mixture pleasantness ratings within the two subgroups separately. The potential of eucalyptol as a masking agent was further analyzed by the calculation of positive and negative correlations between the intensity ratings in the eucalyptol condition (eucalyptol component intensity, ammonia component intensity, and total intensity) and the eucalyptol pleasantness ratings for the whole participant cohort.

### Respiratory data analysis

To explore whether stimulus presentation affected respiration, we examined the respiration volume from a 10-s interval prior (baseline) and subsequent to stimulus application. We subtracted the data “prior” from “subsequent to” stimulus application. This difference was compared including the two independent variables “stimuli” and “group” (pleasant and unpleasant group) in a two-way ANOVA with “difference” as dependent variable. Post hoc tests were Bonferroni corrected and significance thresholds were set to *p* < 0.05.

### fMRI data analysis

FMRI data was analyzed using SPM12 Software (Wellcome Department of Cognitive Neurology) based on Matlab 2017 (MathWorks Inc.). Pre-processing started with slice-timing for temporal correction followed by spatial correction including realignment, co-registration of the anatomical and functional images, segmentation with the help of tissue probability maps, normalization based on deformation fields by means of an MNI-template supplied by SPM12, and smoothing by a Gaussian kernel of 8 × 8 × 8 mm^3^. A general linear model was applied for a within-subject analysis (first level analysis) to convolve individual time courses (onsets and durations of all eight conditions and ratings) and the hemodynamic response function in statistical parametric maps. Individual realignment parameters were added as regressors of no interest. The resulting contrast images were then used for group analysis in a flexible factorial design which automatically creates a subject factor. We combined both experimental sessions in one second-level analysis and included the factor “condition” with four levels (eucalyptol, ammonia, mixture, PG). The main effect of odor was analyzed by contrasting odor and no-odor [(Eu, Am, Mix) vs. PG]. The mixture was supposed to be perceived similarly to eucalyptol, thus, we analyzed differences in the neural activation patterns of the two conditions (Eu vs. Mix; Mix vs. Eu). To explore the effect of the simultaneous presentation of eucalyptol and ammonia in a binary mixture, we grouped the eucalyptol and ammonia conditions and compared them to the mixture condition [(Eu, Am) vs. Mix; Mix vs. (Eu, Am)]. Since the main effect “session” was significant on behavioral level, session-related differences were explored by contrasting the two sessions against each other (Sess1 vs. Sess2; Sess2 vs. Sess1). We repeated the second level analysis for the two subgroups (pleasant and unpleasant group) separately. For the entire fMRI data analysis, activations were considered significant with family-wise-error (FWE) correction *p* < 0.05 for whole-brain comparisons on cluster level and a cluster size of *k* ≥ 5 voxels. The Anatomy Toolbox implemented in the SPM package (Eickhoff et al. 2005) and MRIcron (Rorden et al. 2007) were used for labeling the neuroanatomical structures.

### ROI analysis

To verify the stimulus duration of 4 s, we conducted a region of interest (ROI) analysis using the SPM toolbox MarsBaR (Brett et al. 2002). For this purpose, the piriform cortex was defined as ROI (left and right) based on functional data gained by the contrast all odors > PG. To verify this ROI, we compared it to those coordinates of the piriform cortex published by Seubert et al. (2013). We further analyzed area under the curve to compare the odor conditions excluding PG. Since the data of two out of three conditions were not normally distributed as assessed by the Shapiro–Wilk test, we applied Friedman test and Wilcoxon signed-rank test. Due to multiple comparisons, results were Bonferroni corrected resulting in a significance level set to *p* < 0.017.

## Results

### Behavioral data

*Intensity ratings* In model I, we found three significant main effects: “session” [*F*(1, 1149.2) = 5.3, *p* < 0.05, *η*^2^ = 0.005] indicating that the intensity ratings were higher in session 1 compared to session 2 (session 1: *M* = 40.8, SD = 20.3; session 2: *M* = 38.7, SD = 29.9), “stimulus” [*F*(3, 91,233.1) = 417.9, *p* < 0.001, *η*^2^ = 0.550] indicating that the stimuli were perceived as having varying intensities with mixture as most and ammonia as less intense (mixture: *M* = 55, SD = 23.8, eucalyptol: *M* = 52.7, SD = 30, ammonia: *M* = 36.5, SD = 22.9; PG: *M* = 14.7, SD = 21.8), and “target” [*F*(3, 39,349.5) = 180.3, *p* < 0.001, *η*^2^ = 0.346] indicating that total intensity was rated as more intense than both component intensities (total: *M* = 48.9, SD = 30.3, eucalyptol component: *M* = 36.5, SD = 34.7, ammonia component: *M* = 23.8, SD = 25.3).

The interactions “session” × “stimulus” [*F*(3, 31.2) = 0.1, *p* = 0.934, *η*^2^ = 0.000], “session” × “target” [*F*(3, 77.7) = 0.4, *p* = 0.785, *η*^2^ = 0.001], and “session” × “stimulus” × “target” [*F*(9, 76.4) = 0.4, *p* = 0.958, *η*^2^ = 0.003] were not significant. The interaction “stimulus” × “target” was significant [*F*(9, 33,885.3) = 155.2, *p* < 0.001, *η*^2^ = 0.577].

Pairwise comparisons showed that perceived intensity depended on both what was presented (stimulus) and what was rated (target): Eucalyptol component intensity did not differ between pure eucalyptol and mixture (pure: *M* = 70, SD = 24.2; mixed: *M* = 65.4, SD = 29.9; *p* = 0.444) while ammonia component intensity was perceived as significantly less intense in the mixture compared to pure ammonia (pure: *M* = 52.2, SD = 32.7; mixed: *M* = 33.1, SD = 33.7; *p* < 0.001).

**Fig. 1:**
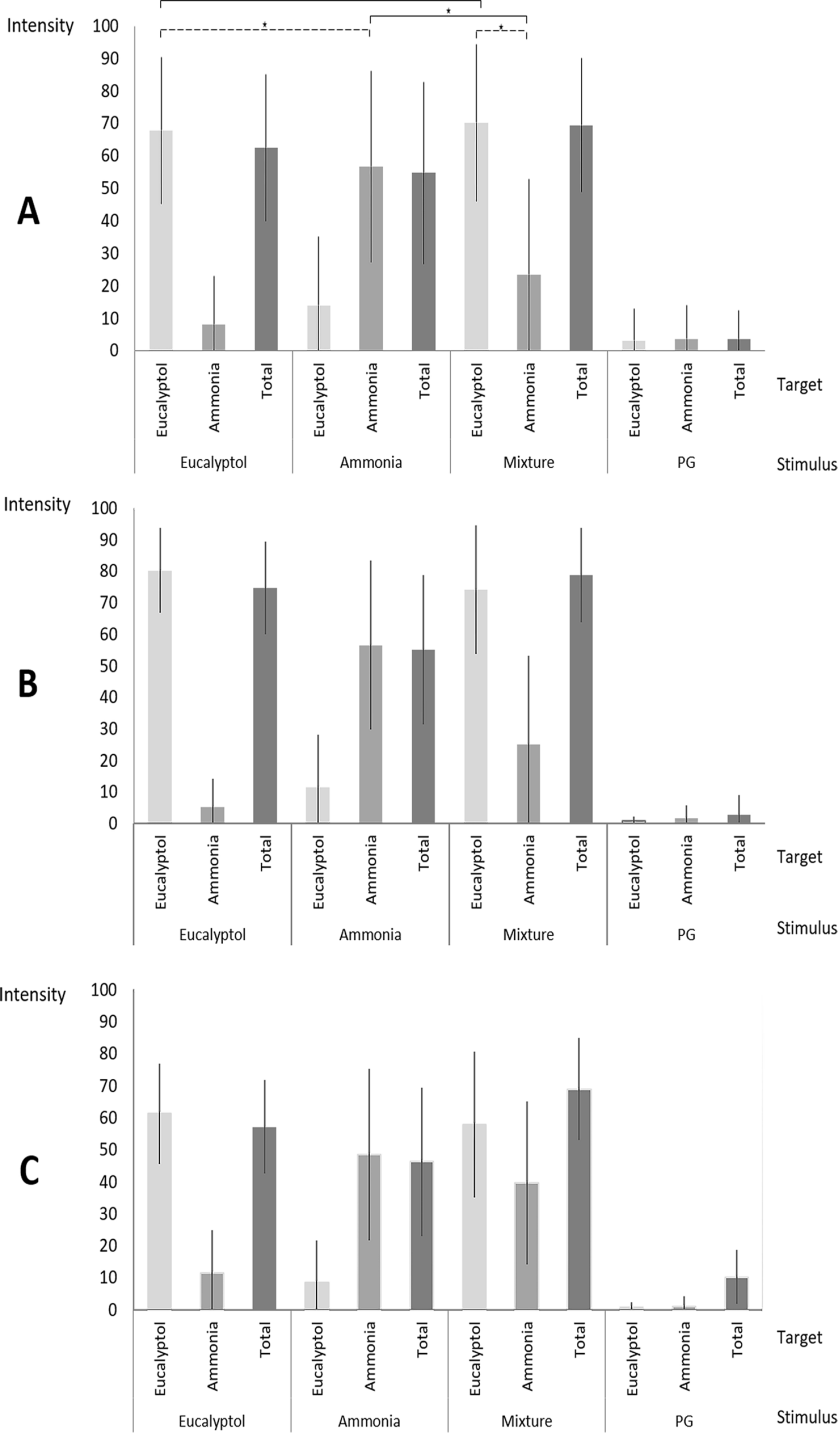
Mean values of the intensity ratings depicted per target (what was rated) and stimulus (what was presented). Bars indicate standard deviations. **a** Data of all participants: relevant pairwise comparisons are marked and significance is illustrated with an asterisk (*p* < 0.05). The interaction “stimulus” × “target” (model I) is indicated by solid lines, the interaction “target” × “stimulus type” (model II) is indicated by dotted lines. Both interactions were significant (*p* < 05). **b** Pleasant group data, and **c** unpleasant group data

In other words, the difference in the eucalyptol component between the pure and mixed odors was significantly smaller than the difference in the ammonia component between the pure and mixed odors (Fig. 1a).

Comparing total intensity ratings, eucalyptol was rated as significantly more intense than ammonia and both were significantly less intense than the mixture (total eucalyptol: *M* = 65.2, SD = 22.4; total ammonia: *M* = 50.3, SD = 29.2; total mixture: *M* = 73.5, SD = 21.9, *p* < 0.001; PG: *M* = 6.8, SD = 16.4;* p* < 0.05).

In model II, we found a significant main effect “stimulus type” [*F*(1, 9269.1) = 23.2, *p* < 0.001, *η*^2^ = 0.083] indicating that intensity was higher in the pure conditions compared to the mixed condition (pure: *M* = 61.1, SD = 21.5; mixed: *M* = 49.2, SD = 26), and “component” [*F*(1, 41,422.7) = 103.8, *p* < 0.001, *η*^2^ = 0.289] indicating that the eucalyptol component was rated as more intense than ammonia component (eucalyptol: *M* = 67.7, SD = 19.8; ammonia: *M* = 42.6, SD = 22.2). The main effect “session” [*F*(1, 1388.8) = 3.5, *p* = 0.06, *η*^2^ = 0.013], the interaction effects “session” × “stimulus type” [*F*(1, 15.1) = 0.04, *p* = 0.846, *η*^2^ = 0.000], “session” × “component” [*F*(1, 31.3) = 0.1, *p* = 0.780, *η*^2^ = 0.000], and “session” × “stimulus type” × “component” [*F*(1, 76.3) = 0.2, *p* = 0.662, *η*^2^ = 0.001] were not significant. The interaction effect “stimulus type” × “component” was significant [*F*(1, 3469.9) = 8.7, *p* < 0.05, *η*^2^ = 0.033]. Post hoc tests revealed that the eucalyptol component was rated as significantly more intense than the ammonia component comparing both the pure odors’ components (eucalyptol: *M* = 70.0, SD = 24.2; ammonia: *M* = 52.2 SD = 32.7 *p* < 0.000) as well as the mixture components (eucalyptol: *M* = 65.4, SD = 29.9; ammonia: *M* = 33.1, SD = 33.7 *p* < 0.001).

This means that the difference between the eucalyptol and ammonia component was significantly higher when both odors were mixed compared to the pure odors (Fig. 1a).

For visualization purposes, intensity ratings per group are depicted in Fig. 1b, c.

**Fig. 2:**
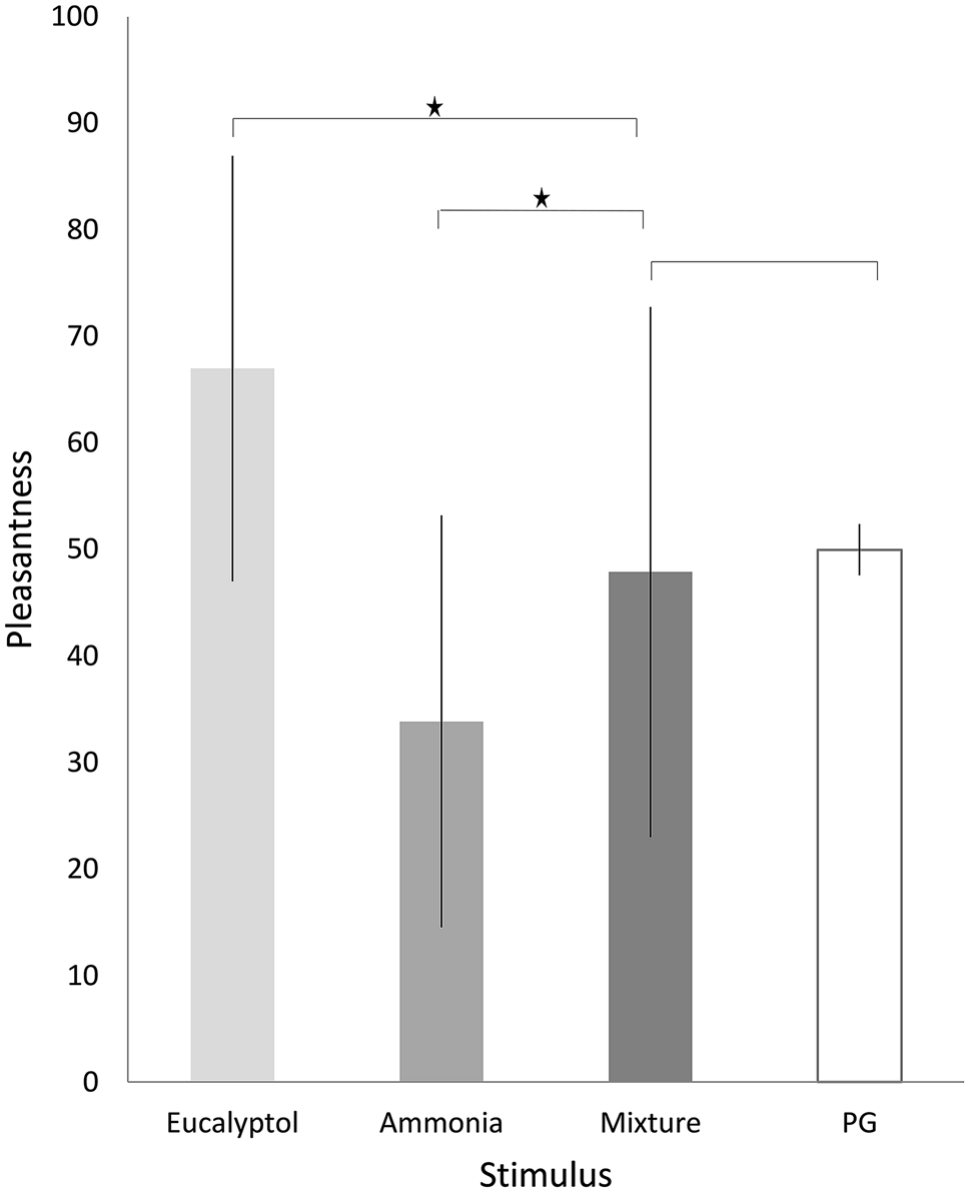
Mean values of the pleasantness ratings per stimulus including all participants. Bars indicate standard deviations and significance is marked with an asterisk (*p* < 0.001)

*Pleasantness ratings* The ratings served to observe how pleasantness changed when the pleasant eucalyptol and the unpleasant ammonia were mixed (Fig. 2). The main effect of “session” [*F*(1, 16.5) = 0.1, *p* = 0.749, *η*^2^ = 0.005) and the interaction “session” × “stimulus” [*F*(3, 2.8) = 0.01, *p* = 0.997, *η*^2^ = 0.000] were not significant which revealed that the ratings did not differ between the sessions. We found a significant main effect of “stimulus” [*F*(3, 12,150) = 75.4, *p* < 0.001, *η*^2^ = 0.550]. Pairwise comparisons yielded that participants rated ammonia as less pleasant (ammonia: *M* = 33.9, SD = 19.3, PG: *M* = 49.9, SD = 2.4, *p* < 0.001) and eucalyptol as more pleasant than non-smelling PG (eucalyptol: *M* = 67, SD = 20; *p* < 0.001). The comparison of mixture and PG did not reach significance (mixture: *M* = 47.9, SD = 24.9, *p* = 1.000).

**Fig. 3:**
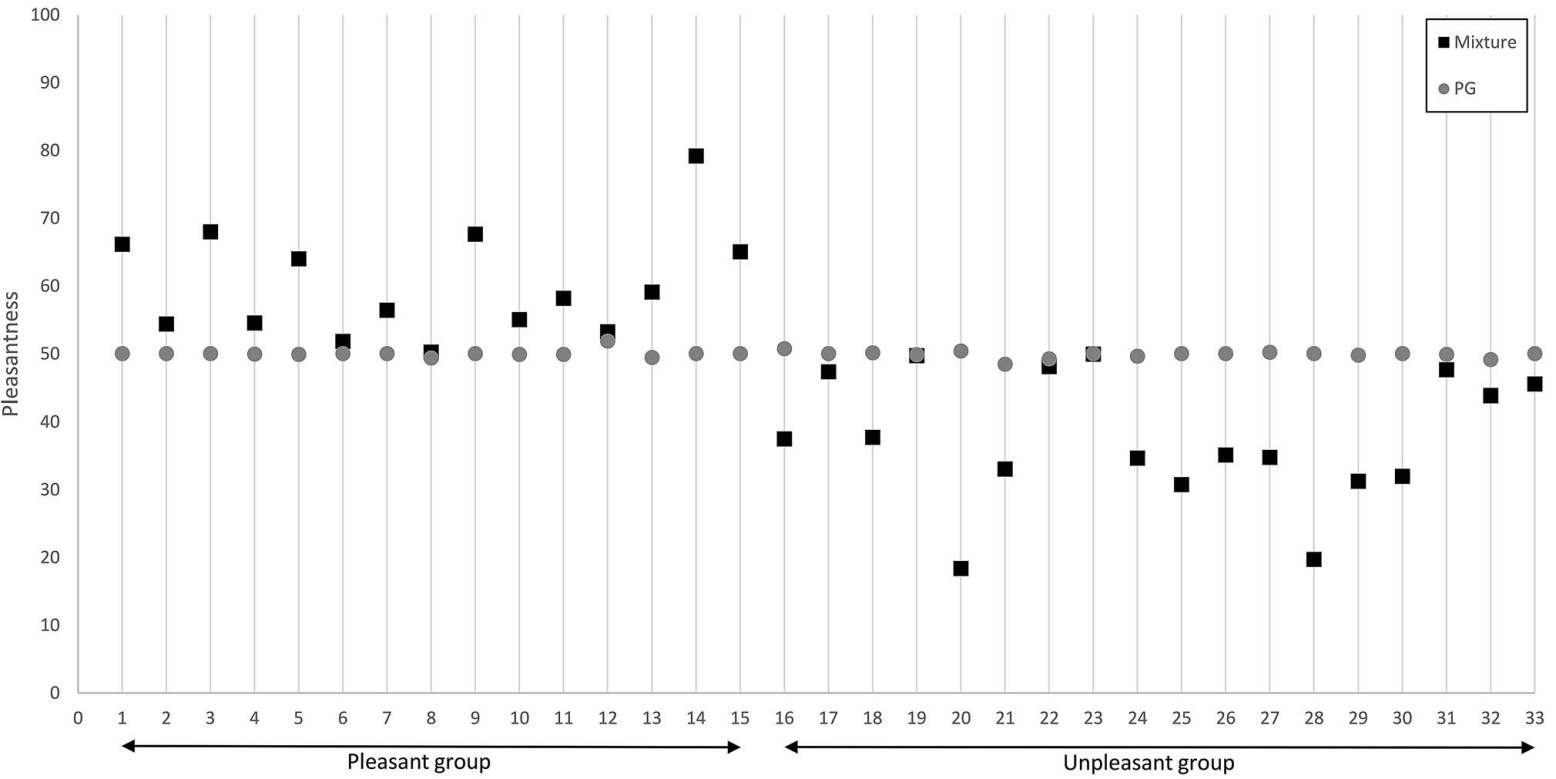
Pleasantness ratings per subject grouped into the pleasant (left) and the unpleasant (right) group for mixture and PG. Numbers on the *x*-axis only enumerate the participants, they do not represent the testing order. The grey dots represent mean values of PG pleasantness, the black squares those of mixture pleasantness

Taking a closer look at the mixture pleasantness ratings per subject as shown in Fig. 3, we observed opposing responses. Fifteen participants (pleasant group) rated the mixture as more pleasant than PG, which means that they scaled it above 50 on the VAS (*M* = 60.2; SD = 7.7) and 18 participants (unpleasant group) scaled the mixture lower than 50 on the scale (*M* = 37.6; SD = 9.3).

A paired sample *t* test revealed significant differences between the groups regarding how intense they perceived the single mixture components: The pleasant group perceived the eucalyptol component significantly more intense in the mixture than the unpleasant group (pleasant group: *M* = 74.2; SD = 17.4; unpleasant group: *M* = 56.2; SD = 19. 5; *p* < 0.05; *t* = 2.3) and the unpleasant group rated the ammonia component as significantly more intense than the pleasant group (pleasant group: *M* = 25.2; SD = 11.1; unpleasant group: *M* = 41.5; SD = 21.4; *p* < 0.05; *t* = − 3.3). The comparison of the total mixture intensity ratings did not reach significance (pleasant group: *M* = 78.9; SD = 10.6; unpleasant group: *M* = 67.8; SD = 17. 2, *p* < 0.05; *t* = 1.9).

Correlation analyses of the intensity and pleasantness ratings of the mixture within each group revealed for the pleasant group a positive correlation between pleasantness and eucalyptol component intensity (*r* = 0.5; *p* < 0.05) as well as total mixture intensity (*r* = 0.5; *p* < 0.05) and no correlation between ammonia component intensity (*r* = − 0.3; *p* = 0.3) and pleasantness. For the unpleasant group, we found a negative correlation between the ammonia component intensity (*r* = − 0.6; *p* < 0.05) and pleasantness but no correlation between pleasantness and the eucalyptol component as well as total intensity (eucalyptol component: *r* = 0.2; *p* = 0.4; total: *r* = − 0.3; *p* = 0.3). Including the entire participant cohort in the analysis, the eucalyptol intensity positively correlated with the pleasantness of the eucalyptol stimulus (eucalyptol component intensity: *r* = 0.5; *p* < 0.05; total intensity: *r* = 0.5; *p* < 0.05).

### Respiratory data

We found no significant main effects of “stimulus” [*F*(3, 122,529.3) = 1.7, *p* = 0.17], “group” [*F*(1, 3991.5) = 0.06, *p* = 0.8] and no significant interaction “stimulus × group” [*F*(3, 3597.1) = 0.05, *p* = 0.9]. Thus, changes in respiratory volumes could not account for possible differences on the behavioral or cortical level. Further information on respiratory data is provided in the supplementary material. Supplementary Fig. 1 visualizes the respiratory volume difference comparing the single odorants and the mixture per group (pleasant and unpleasant).

### fMRI data

**Fig. 4:**
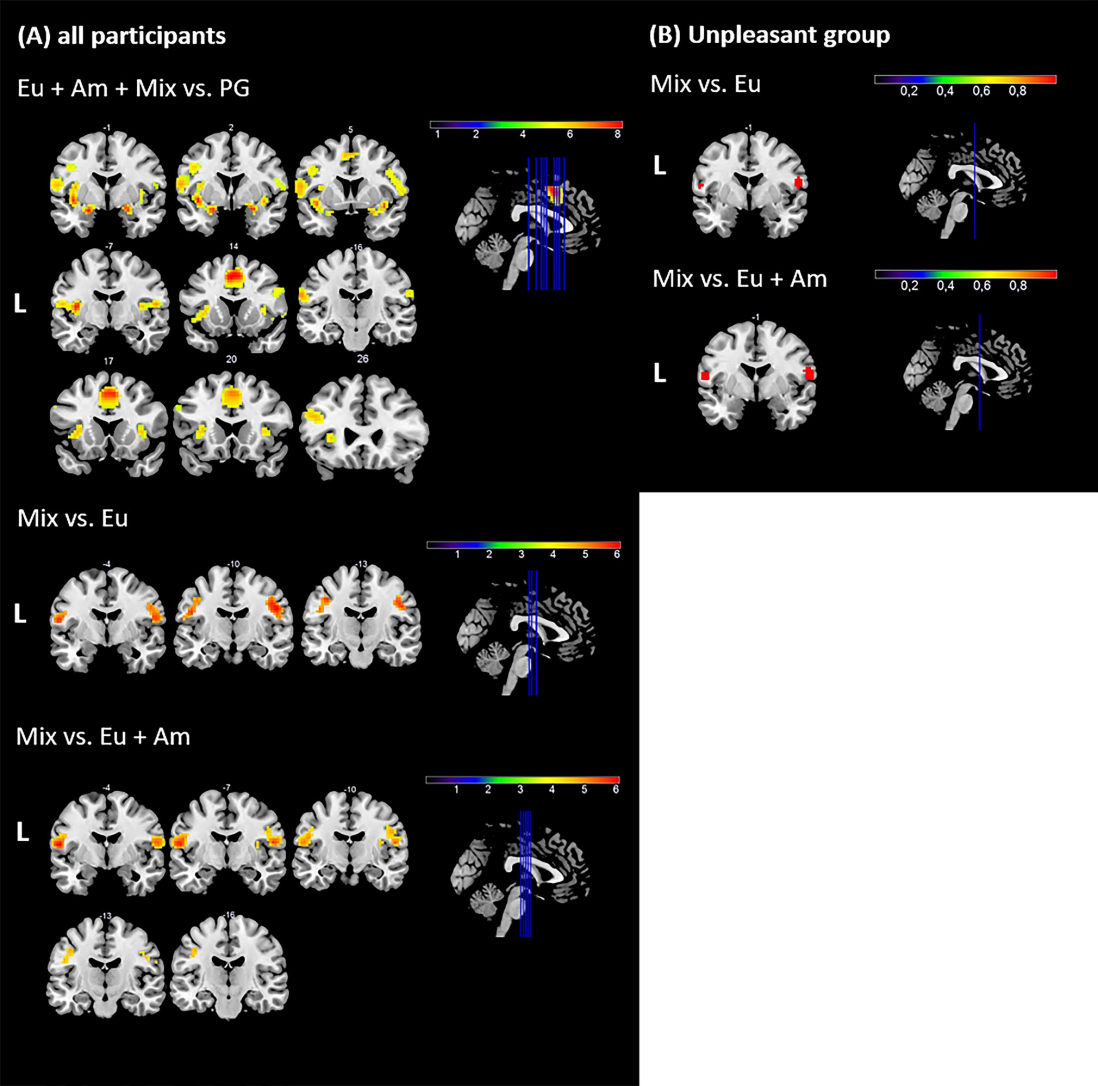
Neural activation in response to (1) all odors, (2) the mixture contrasted to eucalyptol, and (3) the mixture contrasted to eucalyptol and ammonia. A indicates those activations based on the data of all participants and B depicts neural responses in the unpleasant group. Blue lines indicate the locations of the displayed slices and the numbers above the slices indicate stereotactic coordinates in axial orientation. L indicates the left side of the brain. Illustrated data include clusters with a size of *k* ≥ 5 voxels and are FEW-corrected (*p* < 0.05)

The main effect of odor (Eu + Am + Mix vs. PG) revealed neural activation in regions processing chemosensory signals: the left anterior midcingulate gyrus, the bilateral pirC and neighboring amygdala, the bilateral insula including anterior and posterior parts, the right parietal operculum (SII), the left triangular part of the inferior frontal gyrus, the right postcentral gyrus (SII) and in other regions including the left precentral gyrus and the bilateral calcarine gyrus (Table 1a, Fig. 4a). In a next step, we analyzed differences between the activations in response to eucalyptol and mixture stimulation. Using eucalyptol as reference (mix vs. Eu), areas that would be typically activated by olfactory stimulation were omitted. Instead, we observed two bilateral activation clusters including the postcentral gyrus in both hemispheres and the left precentral gyrus (Table 1b, Fig. 4a). The contrast Eu vs. mix did not yield any significant activation. To find regions which were solely activated by a mixture, we contrasted it to the sum of its components (mix vs. Eu + Am). This contrast produced similar bilateral activation clusters as described for the previous contrast (mix vs. Eu). The postcentral gyrus (SI) and the precentral gyrus were activated in the left hemisphere, while the postcentral gyrus (SI), the precentral gyrus, the parietal operculum (SII), and the posterior insula were activated in the right hemisphere (Table 1c, Fig. 4a). For the contrast (Eu + Am) vs. mix, no significant activation was found. Contrasting the two sessions, enhanced left-sided activation in the angular gyrus, the lingual gyrus, and the lentiform nucleus during the first session (Sess1 vs. Sess2; Table 1d) were observed but no activation in the opposite contrast (Sess2 vs. Sess1). We next analyzed brain responses per subgroup (pleasant group and unpleasant group). The first contrast, Eu + Am + mix vs. PG, indicated neural responses in the left insula and the right pirC adjacent to the amygdala for the pleasant group (Table 2a)*.* In the unpleasant group, we found bilateral activation in the rolandic operculum, the pirC and neighboring amygdala regions, the insula including anterior and posterior regions, as well as left-sided activation in the anterior midcingulate gyrus and the postcentral gyrus (SII) (Table 3a). Unlike the pleasant group, the unpleasant group showed bilateral neural activation in the parietal operculum (SII) when comparing mixture and eucalyptol (mix vs. Eu) (Table 3b, Fig. 4b). Similar results were obtained for the last contrast, mix vs. (Eu + Am). In the pleasant group, no activation survived the statistical threshold while the bilateral parietal operculum (SII) and the left posterior insula were activated in the unpleasant group (Table 3c, Fig. 4b). The other comparisons, Eu vs. mix and (Eu + Am) vs. mix, did not yield activation for any of the groups.

**Table 1:** Significant FWE-corrected (*p* < 0.05) neural activations including functional data of all participants (*n* = 33)

Anatomical labels	Cluster size	*T*	MNI coordinates		
			*x*	*y*	*z*
a (Eu + Am + mix) vs. PG					
***L anterior midcingulate gyrus***	269	8.15	− 3	14	47
***L piriform cortex***	536	7.76	− 21	− 1	− 16
***L posterior insula***		7.70	− 39	− 7	11
***L insula***		7.65	− 39	2	− 7
***R piriform cortex and neighboring amygdala***	300	7.44	24	2	− 16
***R insula***		7.02	39	5	− 10
***R parietal operculum (SII)***		6.43	54	− 7	14
***L IFG, pars triangularis***	98	6.18	− 45	26	26
***L IFG, pars triangularis***		4.91	− 57	20	32
***R anterior insula***	32	5.95	36	17	5
***L precentral gyrus (M1)***	45	5.78	− 42	2	35
***R postcentral gyrus (SII)***	17	5.56	63	− 16	26
*** R calcarine gyrus***	15	5.04	15	− 70	11
***L calcarine gyrus***	7	5.01	− 18	− 67	5
* R pallidum*	4	4.81	15	8	2
* R IFG, pars triangularis*	1	4.72	51	38	26
* L calcarine sulcus*	2	4.68	− 6	− 73	11
* R precentral gyrus*	1	4.65	63	5	26
* L globus pallidus*	1	4.60	− 12	8	2
* R calcarine sulcus*	1	4.56	9	− 88	− 1
b: Mix vs. Eu					
***R postcentral gyrus (S1)***	139	6.06	51	− 10	29
***R postcentral gyrus (S1)***		5.50	57	− 4	20
***L postcentral gyrus (S1)***	102	5.59	− 57	− 4	17
***L precentral gyrus (M1)***		5.53	− 39	− 13	41
***L precentral gyrus (M1)***		5.51	− 48	− 10	26
c: Mix vs. (Eu + Am)					
***L postcentral gyrus (S1)***	136	6.69	− 57	− 4	14
***L precentral gyrus (M1)***		5.15	− 39	− 13	41
***L postcentral gyrus (S1)***		495	− 42	− 16	32
***R parietal operculum (SII)***	113	6.20	54	− 7	17
***R postcentral gyrus (S1)***		5.21	51	− 10	29
***R precentral gyrus (M1)***		4.63	42	− 13	38
***R posterior insula (M1)***	7	4.95	36	− 10	14
d: Sess1 vs. Sess2					
***L angular gyrus***	14	5.11	− 45	− 67	41
***L lingual gyrus***	9	4.96	− 15	− 97	− 7
***L lentiform nucleus***	5	4.70	− 12	2	− 10
* L precentral gyrus*	4	4.70	− 42	8	50
* R lingual gyrus*	3	4.68	18	− 82	− 7

Anatomical labels printed in bold type refer to a cluster size of *k* ≥ 5 voxels. Activations occurred in response to (a) all odor stimuli contrasted to the odorless baseline, (b) mixture stimulation contrasted to eucalyptol stimulation, and (c) mixture contrasted to both pure odors. (d) Activations observed in session 1 compared to session 2

**Table 2:** Significant FWE-corrected (*p* < 0.05) neural activations including functional data of the pleasant group (*n* = 15)

Anatomical labels	Cluster size	*T*	MNI coordinates		
			*x*	*y*	*z*
a: (Eu + Am + mix) vs. PG					
***L posterior insula***	15	5.56	− 39	− 7	11
***L insula***	6	5.38	− 39	2	− 4
***R piriform cortex and neighboring amygdala***	5	4.96	24	2	− 16
* L amygdala*	3	5.10	− 21	− 1	− 16
* R anterior midcingulate gyrus*	1	4.81	6	17	44

Anatomical labels printed in bold type refer to a cluster size of *k* ≥ 5 voxels. Activation occurred in response to all odor stimuli contrasted to the odorless baseline

**Table 3:** Significant FWE-corrected (*p* < 0.05) neural activations including functional data of the unpleasant group (*n* = 18)

Anatomical labels	Cluster size	*T*	MNI coordinates		
			*x*	*y*	*z*
a: (Eu + Am + mix) vs. PG					
***L anterior midcingulate gyrus***	103	6.55	− 3	14	47
***L rolandic operculum***	52	5.94	− 60	2	14
***L piriform cortex***	10	5.72	− 21	2	− 19
***L postcentral gyrus (SII)***	15	5.61	− 66	− 19	26
***L anterior insula***	22	5.54	− 30	23	5
***L insula***		4.80	− 39	14	− 1
***L insula***	12	5.47	− 39	2	− 7
***R parietal operculum (SII)***	5	5.26	54	− 7	14
***R piriform cortex and neighboring amygdala***	8	5.10	24	2	− 16
***R anterior insula***	13	5.10	36	17	5
***R rolandic operculum***	7	4.94	60	8	8
***L posterior insula***	7	4.93	− 39	− 7	8
* L inferior frontal gyrus pars triangularis*	3	4.98	− 51	29	29
* R posterior Insula*	1	4.86	39	− 4	8
* R posterior Insula*	2	4.80	39	5	− 7
* R calcarine sulcus*	1	4.78	− 18	− 67	5
* L calcarine sulcus*	3	4.76	− 6	− 76	11
* R inferior frontal gyrus pars opercularis*	3	4.72	48	8	20
* L postcentral gyrus*	1	4.69	− 57	− 10	14
b: Mix vs. Eu					
***L parietal operculum (SII)***	19	5.10	57	− 1	14
***R parietal operculum (SII)***	5	5.08	− 57	− 1	14
* R precentral gyrus*	1	4.71	45	− 10	44
c: Mix vs. (Eu + Am)					
***L parietal operculum (SII)***	41	6.01	− 57	− 4	14
***R parietal operculum (SII)***	43	5.55	57	− 4	14
***R anterior insula***	7	4.85	39	24	8

Anatomical labels printed in bold type refer to a cluster size of *k* ≥ 5 voxels. Activations occurred in response to (a) all odor stimuli contrasted to the odorless baseline, (b) mixture stimulation compared to eucalyptol stimulation, and (c) mixture stimulation contrasted to stimulation with both pure odors

### ROI analysis

**Fig. 5:**
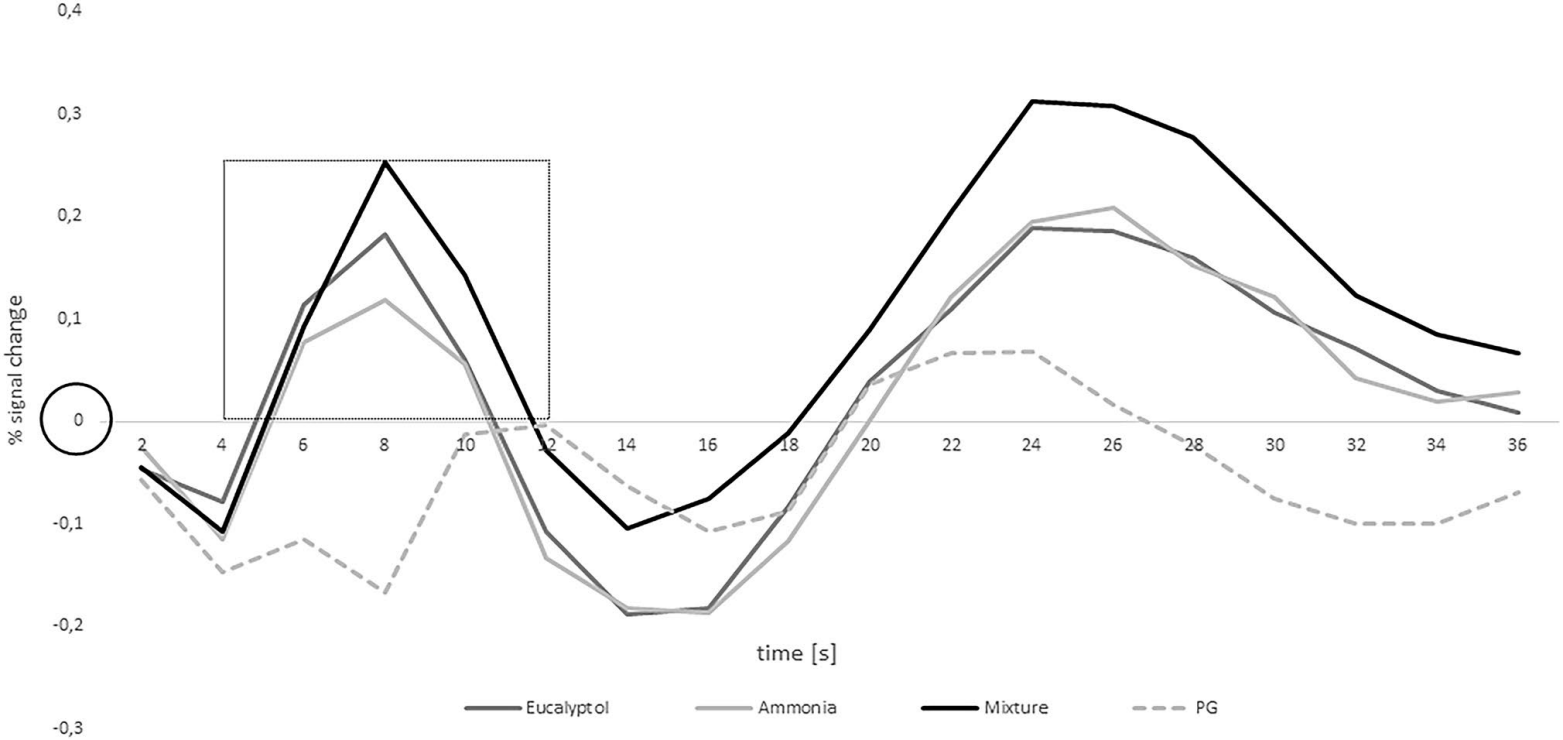
Results of the ROI analysis comparing the four conditions. The black circle marks the odor delivery onset. The box indicates which time frame was considered for area under the curve analysis

ROI analysis showed an increasing response following the olfactory stimulation (Fig. 5). After the initial dip (2–4 s), the peak was reached after 8 s which fits the hemodynamic response function of the BOLD signal. In comparison to the olfactory conditions, the graph representing PG mainly remains below the *x*-axis since piriform cortex is not activated by the odorless baseline. The data of two out of three odor conditions were not normally distributed as assessed by the Shapiro–Wilk test (eucalyptol and mixture: *p* < 0.01; ammonia: *p* = 0.46). Analysis of the area under the curve reached significance in signal change [*χ*^2^(2) = 20.96; *p* < 0.01]. Post hoc analysis revealed a significantly lowest signal change for ammonia compared to eucalyptol and mixture (eucalyptol: *Z* = − 3.0; *p* < 0.017; mixture: *Z* = − 4.6; *p* < 0.017) while there was no significant difference between eucalyptol and mixture (*Z* = − 2.0; *p* = 0.05). These results are in line with the perceived odor intensities reported in the behavioral results section. The reason for the second peak (24 s) depicted in Fig. 5 remains unclear. Analyzing this goes beyond the scope of this paper and should be explored in future research.

## Discussion

In the present study, we explored the potential of eucalyptol as a masking agent to cover the unpleasant odor component in ammonia-containing products. Essential requirements for malodor coverage are an improved olfactory sensation and a pleasant percept of the final mixture. We repeated the results of our recent study that eucalyptol can serve as a suitable tool to mask the smell of ammonia, which was based on the finding that mixing affected the ammonia component significantly more than the eucalyptol component. These results are discussed in detail in the previous study (Müschenich et al. 2019). Further results are discussed in the following:

### Disagreement among the participants: successful masking in the pleasant group but SII activation in the unpleasant group

Inconsistent with previous research (Frasnelli et al. 2011; Grabenhorst et al. 2007; Lawless 1977), our mixture was perceived very differently based on the individual so we grouped the participants into a pleasant and unpleasant group. This contradicts our first hypothesis that the mixture was as pleasant as eucalyptol. To control for this effect on a cortical level, we contrasted neural activation patterns in response to the mixture and the pure eucalyptol in both groups separately (mix vs. Eu). In the pleasant group, no activation was shown to be statistically significant. Both stimuli seemed to be processed and perceived equally, and the masking was successful. In the unpleasant group, we found bilateral activation in the second somatosensory cortex (SII). This is in line with findings by Croy et al. (2016): The unpleasant mixture perception could have enhanced participants’ susceptibility to the painful intranasal trigeminal percept of the mixture represented in SII activation.

### What are these group differences based on?

Mixture perception may depend on whether the mixture is processed elementally (perception of the single mixture components) or configurally (mixture perception as a single odor). Thus far, little is known whether only one processing strategy is engaged or both occur simultaneously (Howard and Gottfried 2014; Jinks and Laing 2001). Recent studies found the concentration ratio of mixture components, participants’ motivation, and attention as crucial factors to determine which strategy is applied (Romagny et al. 2018; Sinding et al. 2013). It has to be kept in mind, that ammonia is an unpleasant, trigeminal, and potentially threatening stimulus. Therefore, it is likely that attention was rather focused on the ammonia component, at least in the unpleasant group. Hypothetically, this group was less experienced with ammonia than the pleasant group. Thus, the participants’ sensory processing focused more on the ammonia component, further increasing its unpleasantness (Miron et al. 1989). In contrast, the pleasant group could have been more experienced with ammonia leading to reduced attention towards the mixture. It was also proven that memorizing a stimulus, here eucalyptol, can inhibit the unpleasant impact of another one, which could be the case in the pleasant group (Bensafi et al. 2012; Jinks and Laing 2001; Rozin et al. 1982). These considerations regarding group differences are highly speculative and need to be further investigated by asking participants to rate familiarity in future studies. In this regard, it has to be considered that perceived intensity and pleasantness might affect each other. However, our results show that strength and direction of the correlation between perceived intensity and pleasantness depend on the presented stimulus. Thus, the halo-effect is rather unlikely. Nevertheless, we suggest to randomize the order of ratings in future studies.

On neural level, we provide evidence for the anterior insula’s role in focusing participant attention towards a certain mixture compound. Contrasting mixture activation to the grouped activation of the single odorants, we found activation only in the unpleasant group (Brooks et al. 2002; Peyron et al. 1999). This supports the hypothesis that this group paid more attention towards the mixture since they assessed the ammonia component as a relevant warning signal. Insular activation could reflect the enhanced perceived salience, unpleasantness or pain-intensity of this group which could be further reasons for an attentional shift (Bensafi et al. 2012; Coghill et al. 1999; Grabenhorst et al. 2007; Moessnang et al. 2013). As activation in the insular cortex and SII are strongly correlated, they were often referred to as operculo-insular cortex (Baumgärtner et al. 2010; Lötsch et al. 2012; Peyron et al. 2002). Activation here was associated with salient event detection and responding to behaviorally relevant stimuli such as ammonia within a mixture (Hummel et al. 2013; Legrain et al. 2011; Lötsch et al. 2012). In the pleasant group, we expected that the addition of ammonia would at least cause unconscious processing (Grabenhorst et al. 2011) but no activation survived.

### The halo-dumping effect could serve as an explanation

Olfactory activation did not reach statistical threshold in any of the groups which could be explained by a previously observed suppression of the olfactory signal by the trigeminal component (Boyle et al. 2007a, b). With regard to the two groups, the reason for the observed group differences could be attributed rather to the trigeminal system. One of the core implications of our previous study (Müschenich et al. 2019) was an enhanced trigeminal sensation of the mixture compared to the pure odors, likely, leading to a more unpleasant, even painful mixture sensation. Since we used the identical experimental setup including stimuli and duration in the present study, we can adopt the results. Thus, trigeminal pleasantness and individual sensitivity towards trigeminal stimulation have to be considered here. Sensitivity could vary between individuals due to nasal anatomy (Konstantinidis et al. 2010; Scheibe et al. 2014) and there were inconsistent results regarding gender-related differences (Hummel et al. 2003, 2007; Lundström et al. 2005). The factor gender was balanced in our groups but we did not control for trigeminal sensitivity prior to participation. Stankewitz et al. (2010) for example, chose their participants based on how sensitive they responded to trigeminal stimulation. To overcome this, we chose the ammonia concentration of 60 ppm which is clearly above olfactory and trigeminal threshold defined by Smeets et al. (2007). To provide a masking effect, we did not use higher ammonia concentration. This opens possibilities to investigate mixture processing without masking effects mixing different ammonia and eucalyptol concentrations.

Unlike the above-mentioned olfactory suppression by the trigeminal system, we found olfactory mixture enhancement on behavioral level. Eucalyptol and ammonia seem to intensify each other. Both olfactory and trigeminal enhancement could be based on the halo-dumping effect which is an important consideration in this respect (Clark and Lawless 1994). With regard to the present study, this means that the olfactory and trigeminal percept affected each other and that the rated intensity as well as the pleasantness reflect both percepts in one attribute. Since it is most common to perceive odors as an interplay between the olfactory and trigeminal system in everyday life, we did not differentiate between the two concepts and our study serves as a holistic approach. However, we cannot exclude undetected interactions between the two sensory modalities in our paradigm. Such a differentiation remains an interesting question for further research investigating correlations between perceived trigeminal and olfactory intensity and pleasantness separately and the corresponding underlying neural correlates.

### Including all participants revealed further activation in the pain matrix

Activation in the anterior insula and SII found in the unpleasant group indicated that the mixture stimulated parts of the pain matrix processing noxious and salient input (Albrecht et al. 2010; Baumgärtner et al. 2010; Iannilli et al. 2007; Jensen et al. 2016; Legrain et al. 2011; Peyron et al. 2000). Due to enhanced statistical power when the data of all participants was included, stronger activation and additional regions of the pain matrix, namely the primary motor and somatosensory cortex (MI, SI), were found in response to the bimodal mixture compared to one or both components (Albrecht et al. 2010; Apkarian et al. 2000; Casey et al. 1996; Gelnar et al. 1999; Jensen et al. 2016; Kollndorfer et al. 2015; Misra and Coombes 2015; Stankewitz et al. 2010). These activations provide further evidence for an increased intensity leading to a painful mixture percept. Thus, processing differences are rather related to the trigeminal sensation while there was no activation in regions coding for pure olfactory stimulation such as the OFC or pirC. Since the reversed contrast ((Eu + Am) vs. mix) yielded no activation, we conclude that processing a bimodal mixture is supra-additive and more complex than the linear summation of the components’ neural responses. This contradicts our second hypothesis about similar processing of eucalyptol and mixture.

### Conclusion: what are the implications for eucalyptol as a potential mask?

It was reported that higher odor intensity can lead to less pleasantness depending on concentration range and quality of the respective odorants (Cometto-Muñiz and Cain 1984). We found that the more intense eucalyptol was perceived as pure odor or as mixture component, the more pleasant the entire stimulus was. Thus, eucalyptol could work as an olfactory masking tool. However, we provide evidence for a strong variation between participants which could lead to an unpleasant mixture perception, at least when ammonia was used as malodor. We observed no mutual inhibition of the trigeminal sensations between eucalyptol and ammonia. In contrast, trigeminal enhancement further impeded the masking approach. To allow for a direct comparison with our results, fMRI studies are needed to approach masking behavior of a mixture consisting of olfactory-trigeminal odors.

## Electronic supplementary material

Below is the link to the electronic supplementary material.

Supplementary file1 (DOCX 60 KB)
