# Supplementary material for: Some like it, some do not: behavioral responses and central processing of olfactory–trigeminal mixture perception
Source: Brain Struct Funct. 2020 Dec 23;226(1):247–61. doi: 10.1007/s00429-020-02178-4 (PMC7817597; doi:10.1007/s00429-020-02178-4)
Supplement: Supplementary file 1 — Supplementary file1 (DOCX 60 KB) [file 429_2020_2178_MOESM1_ESM.docx]

SUPPLMENTARY MATERIAL

In order to control for an effect of stimulus presentation on respiration, we analyzed the respiratory volume following the four stimuli eucalyptol, ammonia, mixture and PG. As described in the results section, there was no significant difference between the conditions. For further visualization, we provide two diagrams in the following comparing the mixture to eucalyptol and ammonia, respectively: mixture: *M* = -.11 ± .25, eucalyptol: *M* = -.07 ± .25, *p* = 1.0; ammonia: *M* = .00 ± .28, *p* = .6

***p = 1.0***


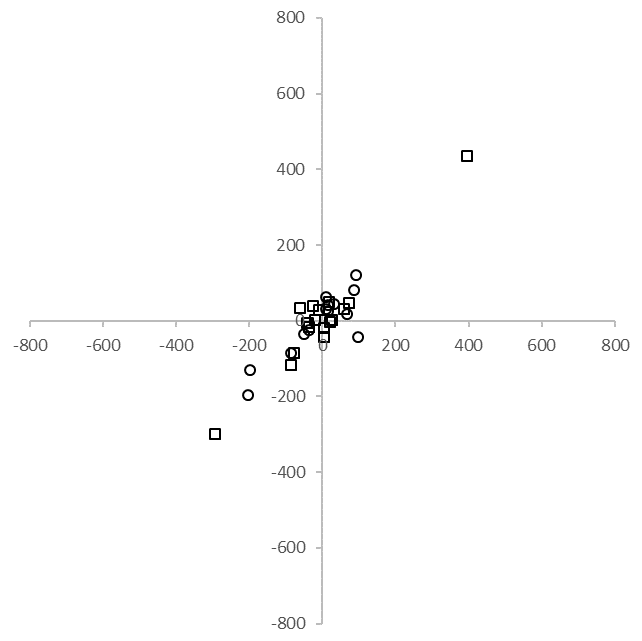


A

Mixture [V*s]

Eucalyptol [V*s]

Mixture [V*s]


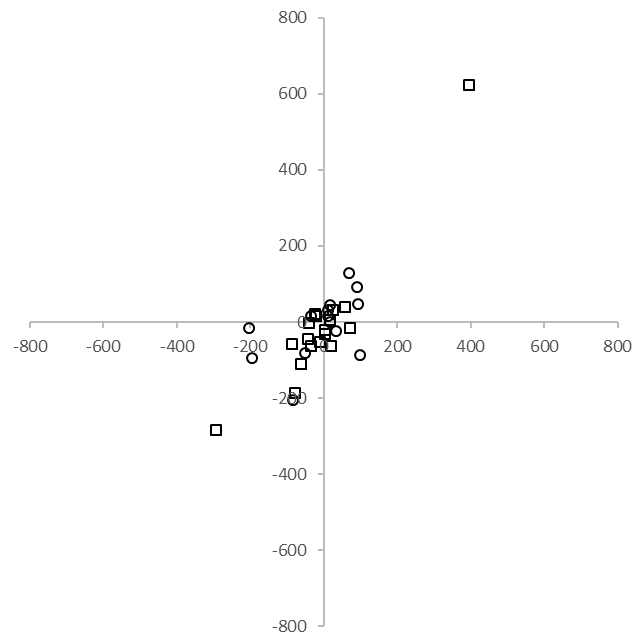


Ammonia [V*s]

B

***p = .6***

**Supplementary Figure 1:** Respiratory volume differences of the single odorants eucalyptol (A) and ammonia (B) as a function of the difference of the mixture stimulus. The pleasant group is represented by the dots (mean value per participant). The unpleasant group is represented by the squares (mean value per participant) and the dashed line depicts the unit slope line. As depicted, respiratory volume is not different between eucalyptol (A) and the mixture (*p* = 1.0) nor between ammonia (B) and the mixture (*p* = .6).
